# Estimation of potential soil erosion in the Prosecco DOCG area (NE Italy), toward a soil footprint of bottled sparkling wine production in different land-management scenarios

**DOI:** 10.1371/journal.pone.0210922

**Published:** 2019-05-01

**Authors:** Salvatore E. Pappalardo, Lorenzo Gislimberti, Francesco Ferrarese, Massimo De Marchi, Paolo Mozzi

**Affiliations:** 1 Department of Civil, Environmental and Architectural Engineering (ICEA), University of Padua, Padua, Italy; 2 Department of Historical and Geographic Sciences and the Ancient world (DiSSGeA), University of Padua, Padua, Italy; 3 Department of Geoscience, University of Padua, Padua, Italy; CSIRO, AUSTRALIA

## Abstract

Agricultural lands are the widest Human-modified ecosystems, making crop production the most extensive form of land use on Earth. However, in conventional agricultural land management, soil erosion may be boosted up to 1–2 orders of magnitude higher than the natural rates of soil production, making unproductive about the 30% of the world’s arable. Nowadays in Europe, vineyards represent the most erosion-prone agricultural lands, especially in Mediterranean countries, showing the highest erosion rates in comparison to other type of land uses. Prosecco wine is produced in NE Italy by a rate of 400 M bottles per year, with the fastest growing demand in the global market at present. A production of 90 M bottles year^-1^ is currently running in the historical Prosecco DOCG (215 km^2^), in a steep hilly landscape of Veneto Region (Conegliano-Valdobbiadene). To sustain wine production, agricultural intensification is at present increasing, by re-setting of hillslopes and land use changes towards new vineyard plantations. The aim of this study is to estimate and to map potential soil erosion rate, calculating a sort of “soil footprint” for wine production in different agricultural land-management scenarios. RUSLE model was adopted to estimate potential soil erosion in Mg ha^−1^ year^−1^, by using high resolution topographic data (LiDAR), 10 years rainfall data analysis, detailed land use and local soil characteristics. For a conventional land-management scenario the estimated that total potential soil erosion in the Prosecco DOCG area is 411,266 Mg year^-1^, with an erosion rate of 19.5 Mg ha year^-1^. Modelled soil erosion is mainly clustered on steep slopes, with rates higher than 40 Mg ha^-1^ year^-1^. In Prosecco vineyards potential soil erosion could reach 300,180 Mg year^-1^, by a mean rate of 43.7 Mg ha^-1^ year^-1^, which is 31 times higher than the upper limit of tolerable soil erosion threshold defined for Europe. In contrast, simulation of different nature-based scenarios (hedgerows, buffer strips, and grass cover) showed soil erosion could be effectively reduced: a 100% inter-row grass cover showed a reduction of almost 3 times in vineyards (from 43.7 to 14.6 Mg ha^-1^ year^-1^), saving about 50% of soil in the whole Prosecco DOCG. The soil footprint modelled for a conventional land-management scenario is about 3.3 kg every bottle produced; in contrast it would be reduced to 1.1 kg/bottle in the completely green land-management scenario. This study, as the first estimation of potential soil erosion at Prosecco DOCG scale, suggests that an integrated and public soil erosion monitoring system is strongly needed in viticultural area, by implementing direct/indirect field measures with spatial analyses at agricultural landscape scale.

## Introduction

### Agricultural lands and soil erosion

Agricultural lands presently occupy about 37.4% (56.1 M km^2^) of the 150 M km^2^ of Earth land surfaces [1]. They amount to the 50% if glaciers, deserts, rocks, and other physical environments not suitable for agriculture are excluded [2–4]. Indeed, agricultural lands are the widest Human-modified ecosystems, making crop production the most extensive form of land use on Earth [5]. The geographical dimension at global scale of agriculture is crucial to understand the role it plays in terms of land degradation and erosion processes, which are boosted up to 1–2 orders of magnitude greater than the natural rates of soil production [6]. In fact, high erosion rate in conventional farming are mainly linked to unsustainable soil management and agricultural practices: intense tillage, soil compaction due to the use of heavy machinery, down-slope cropping on hillside, and intensive herbicide application [7,8]. Recently, it has been estimated that soil erosion directly linked to mismanagement of agricultural lands affects about 5,520,000 km^2^ worldwide [7]. As results of heavy soil erosion, about 30% of the world’s arable land have been already lost and turned to unproductive [9].

In Europe 12.7% of total land surface is affected by moderate to high soil erosion risk [10]. This means that a total area of about 14 M ha (a surface wider than Greece), loses soil at a rate of 2.46 Mg ha^-1^ yr^-1^ on average, resulting in a total annual soil loss of 970 M Mg [11,12]. According to estimation based on erosion plot data, the mean erosion rate of total surface in Italy is 2.3 Mg ha^-1^ yr^-1^, which represents the 12.5% of the total European erosion [13]. Due to unsustainable agricultural practices of intensive crop production, soil erosion is one of the main environmental concern in many sectors of Southern Europe, especially in sloping rainfed croplands. Many field-based researches performed in Spain demonstrated that agricultural practices based on herbicides and conventional tillage results in high erosion rates: Gomez et al. (2003) found that on slopes up to 20% soil erosion could reach 80 Mg ha^-1^ yr^-1^ [14]; Ramos et al. (2008) measured soil profile lowering due to particle detachment of up to 0.2 ± 0.1 mm yr^−1^ along slopes ranging from 2 to 45% in an orchard conventionally tilled [8,15]; Cerdà et al. (2009) found that soil erosion rates in citrus orchards plantations were 2 Mg ha^-1^ after 1 hour of a 5-year return period rainfall thunderstorm [16].

Among agricultural lands, vineyards cover about 76,000 km^2^ of the Earth surface, an area wider than Ireland, mainly oriented to wine production [17,18]. However, about half of world vineyards surfaces is cultivated in Europe (33,000 km^2^) whose 30% is mainly concentrated in Italy (6,950 km^2^), Spain (9,670 km^2^), and France (7,870 km^2^). Vineyards are respectively 2.3%, 1.9%, and 1.2% of the country area [17,18]. Aside from representing one of the most important cultivations in terms of local economies, income, and employment, vineyards recently gained an increasing attention since it is one of agricultural land use that causes the highest soil erosion rates [19,20].

### Soil erosion in Mediterranean vineyards

Due to geomorphological, climatic, and edaphic conditions together with anthropogenic factors, vineyards in Mediterranean ecosystems are particularly inclined to land degradation and soil erosion [21,22]. Agricultural lands for vineyards are often located on hilly areas, on steep slopes, resulting in the highest measured soil erosion compared to rainfed cereals, olives groves plantations or scrublands. In fact, topography is one of the dominant factor affecting soil erosion. In addition, Mediterranean vineyards have to face high intensity rainfall events, mainly concentrated in Spring and Autumn [23]. As well documented, soil erosion processes are strongly influenced by the high magnitude—low frequency rainfall events which presently have to be even more considered in the climate change scenarios [21,24]. Furthermore, Mediterranean lands are generally poor in nutrient and organic matter content which are key factors on soil stability and erodibility [25].

According to all studies published in the last five decades on different conventional vine croplands worldwide, Italian vineyards seems to show the highest soil erosion rate, by an average rate of 40 Mg ha^-1^ yr^-1^ [20]. Here, different studies were performed in conventional vineyards located in hilly regions, both using direct plot-scale measures methods (botanical benchmarks, poles, rainfall and runoff simulations) and GIS modelling techniques: in the “Chianti Classico” viticultural region (Tuscany, Central Italy), the average measured soil losses in conventional vineyards were 42.1 Mg·ha^−1^·yr^−1^[26]; different studies were also performed in erosion plots of NW Italy, where Tropeano (1984) directly measured rates of 47–70 Mg ha^-1^ yr^-1^ [27], while Biddocu et al. (2015) observed a yearly erosion rate ranging from 10.4 to 24.8 Mg ha^-1^ yr^-1^ in conventional and reduced tillage land management respectively [28]; in Sicily, by a nine-year monitoring system based on in-field pole erosion markers yearly erosion rate range from 86 to 118 Mg ha^-1^ yr^-1^ in conventionally tilled vineyards with 16% slope [21].

High erosion rates in conventional viticulture is often related to the market-driven farming intensification of Mediterranean vineyards for wine production, which results in unsustainable soil management. In fact, common practices are mainly based on deep mechanical tillage and chemical weeding without tillage. Both soil management systems result in bare soil during most of the year, leaving wide areas exposed to the rainfall, with a notable increase in runoff and soil erosion rate [29,30].

### Prosecco DOCG wine production

The international wine trade in 15 years grew by 75% in volume and doubled in value, leading in 2015 to a total volume of import equal to 98 million hectoliters [31]. Considering the last five years, with the exception of Champagne, sparkling wine continued to grow with an annual rate of 7% in value and 6% in volume, turning the Prosecco to an emblematic case as one of the most exported in the world [31,32]. Specifically, Prosecco wine production boosted from 2009 after the “Protected Designation of Origin” (PDO) by labelling the Controlled Denomination of Origin (DOC), and the Controlled and Guaranteed Denomination of Origin (DOCG) areas to identify two specific growing areas. In the last decades, the Prosecco wine production has notably increased in the DOCG area due to a combination of global market demand and large investments in the region, which boosted both crop production and land use change into vineyards croplands [33–35]. In 2017, the Prosecco DOCG growing area was officially enrolled in the tentative list the for the UNESCO World Heritage status [32,36]. However, the UNESCO candidacy was criticized both at academic and at civil society levels due weaknesses in terms of the socio-environmental unsustainability of Prosecco farming system [34].

The Prosecco DOCG vineyards increased from some 4,000 ha in 2000 to 5,700 ha in 2010, and well beyond 7,000 ha officially declared in 2016 [34,36–38]. In the annual report of Consortium Conegliano-Valdobbiadene District in just four years (2013–2016) more than 1,000 ha of land were converted to productive vineyards [39–42].

Nowadays, Prosecco wine production is over 400 M and 90 M bottles respectively in the DOC and DOCG geographical areas [43]. According to the official regional policy document, the actual maximum allowed grape yield for Prosecco DOCG production is 13.5 Mg ha^-1^ (Veneto Region, “Disciplinare di produzione dei vini a Denominazione di Origine Controllata e Garantita” (G.U. 173, 2009/07/28; G.U. 183, 2014/08/08) [43,44]. By the actual 90 M bottles production trend, the Prosecco DOCG annual yield seems to have already reached the production limit.

In such viticultural production context, the economic and production factors are driving drastic changes in land use, undermining an ecosystem stability based on soil system, and fueling the debate about the sustainability of vineyards cropland [34–36,45]. Moreover, the rapid expansion of new vineyards and unsustainable intensification of agricultural practices are triggering several territorial conflicts in the Prosecco DOCG area [46].

The general aim of our study is to map and to estimate potential soil erosion at landscape scale in the Prosecco DOCG growing area, calculating a sort of “soil footprint” for bottled wine production. Specific aims are: i) to estimate the potential total soil erosion; ii) to identify the most critical areas in term of soil erosion rates; iii) to simulate different nature-based land management scenarios to reduce soil erosion processes and off-site impacts, applying possible mitigation measures at field scale.

Considering the complexity of the phenomenon and its implications at socio-economic and environmental level, there is an urgent need for a first estimation of the amount, as much as the potential rate of soil erosion, at agricultural landscape scale. Furthermore, modelled erosion rate at a very detailed scale and simulated nature-based scenarios would represent a scientific contribute to support and design sustainable land management for wine production, especially in sensitive areas where soil erosion could be over the tolerable threshold [11].

### The Prosecco DOCG viticultural area

**Fig 1:**
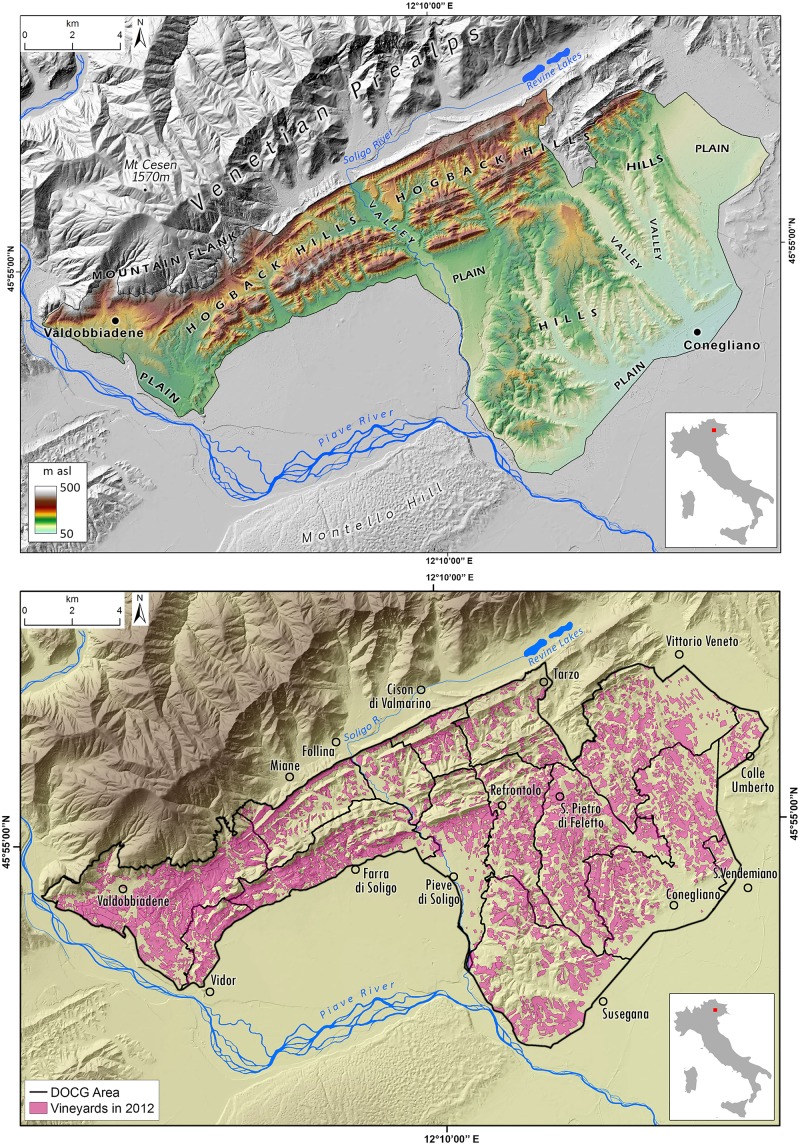
(A) Vineyards distribution in the Prosecco DOCG area. (B) Geographical and geomorphological setting of the Prosecco DOCG area.

**Fig 2:**
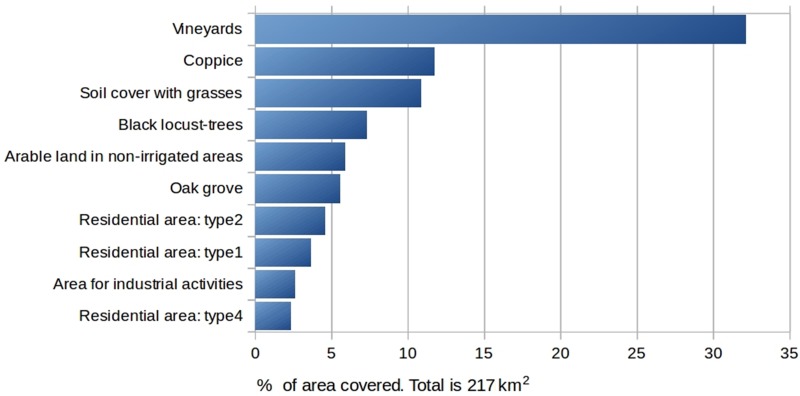
Percentage of area covered by principal land use classes in the Prosecco DOCG zone. More of 30% of DOCG territory is covered by vineyards.

The study area is geographically defined by the Prosecco DOCG wine production region, which spans 215 km^2^ in the North-East sector of Italy (Province of Treviso), and it encompasses fifteen small-medium Municipalities, in a scattered urban-agricultural territorial matrix [34,35]. Vineyard cropland presently occupies the 32% of the DOCG area, representing one of most diffuse cultivation (Figs 1a and 2).

The landscape has elevation ranging from 60 to 500 m a.s.l., mainly dominated by 70% of hilly terrain, and 28% of alluvial plain, while only 2% is mountainous (Fig 1b). The wide and fragmented agricultural landscape is currently dominated by intensive Prosecco cropland (about 86% of the whole cropping system—Figs 1b and 2) which is extended both in the upper alluvial plain and in the hilly areas, which are often scarcely accessible and have steep slopes. In the hilly region, modifications in geomorphology and, therefore, changes in the drainage systems, are often related to crop production intensification and to the high levels of mechanization and standardization required; hence, modern hydraulic-agrarian layouts by vertical ploughing with vineyard rows setup along the steepest slope are now often preferred. On the contrary, contour farming by traditional or modern agricultural terraces are limited, and have been substantially reduced in the past years [34–36].

According to Köppen climate classification, the Prosecco DOCG area is at the transition between a temperate oceanic climate (Cfb) and a Mediterranean type with hot dry-summer (Csa). According to Thornthwaite (1948) climate classification is B3 Humid (60–80 moisture index). Mean precipitation is 1,200 mm yr^-1^ and average temperature is 12.7° C.

Bedrock in the study area spans from Mesozoic dolostone and limestone to Upper Miocene conglomerates (Conglomerato del Montello), sandstone and marls [47].

Fourteen landscape-soil units characterize the study area, ranging from fans, alluvial terraces and valley fills by Prealpine streams of the Last Glaciation with leached soils (C1), to steep hillslopes in conglomerate, with shallow and poorly developed soils (H1), and to long and steep mountain slopes in well-stratified, moderately resistant limestone, with moderately deep and leached soils with clay illuviation (V2) [48]. All landscape-soil units of Prosecco DOCG area are listed in Table 1.

**Table 1:** The landscape-soil units in the Prosecco DOCG area (after ARPAV, 2008).

Landscape and soils characteristics	Landscape-Soil Unit
Fans, alluvial terraces and valley fills by Prealpine streams of the Last Glaciation with carbonate-depleted soils and clay accumulation at depth.	(C1)
Same landforms as C1 with poorly developed soils showing no carbonate depletion	(C2)
Gravelly plain of the Piave River with carbonate-depleted and rubified soils with clay accumulation	(P1)
Same landforms as P1 with carbonate-depleted soils	(P2)
Same landforms as P1 with poorly developed soils and incipient carbonate depletion	(P6)
Fine-grained alluvial plain of the Monticano and Meschio rivers, with poorly developed soils and incipient carbonate depletion	(M3)
Terminal moraines older than the Last Glaciation with moderately thick, carbonate depleted and rubified soils with clay accumulation	(G1)
Terminal moraines of the Last Glaciation with moderately developed, thin soils	(G2)
Steep hillslopes in conglomerate bedrock, with thin and poorly developed soils	(H1)
Low-gradient hillslopes in conglomerate bedrock, with strongly carbonate-depleted, rubified soils with clay accumulation	(H2)
Steep hillslopes in sandstone bedrock, with moderately thick and moderately developed soils	(H3)
Low-gradient hillslopes in marls and siltite bedrock, with moderately thick and moderately developed soils	(H4)
Long and steep mountain slopes in massive and hard limestone, with thin and poorly developed soils	(V1)
Long and steep mountain slopes in well-stratified, moderately resistant limestone, with moderately thick, carbonate-depleted soils with clay accumulation	(V2)

## Material and methods

### RUSLE model

In the past different models and field-based approaches were developed to estimate and to measure soil erosion [20]. Among them, the use of empirical models combined with spatial data processed into Geographical Information Systems (GIS) is the widest tool to quantitatively estimate and map soil erosion rates at basin and landscape scales [49]. To estimate soil erosion in the study area, we adopted the Revised Universal Loss Equation (RUSLE) defined by Renard *et al*. [50], and derived from the Universal Soil Loss Equation (USLE), previously proposed by Wischmeier and Smith (1978) [51]. RUSLE is the most widely-used empirical model for soil erosion estimation at landscape scale [9,12,52,53]. It was also tested in several study cases in Mediterranean context, both at basin and landscape scale [28,54–56]. Moreover, in European Union RUSLE model are presently used to assess land-management scenarios, incorporating mitigation measures such as the Good Agricultural and Environmental Condition (GAEC) of the Common Agricultural Policy (CAP) [10,57].

RUSLE model is based on the main factors which strongly contribute to soil erosion processes, combining data about topography, soils, rainfall, and land use in a GIS environment. It performs a spatial simulation of the erosion processes estimating soil erosion in terms of Mg ha^-1^ yr^-1^. According to quality and geometric resolution of spatial data, by running the RUSLE model it is possible to identify the magnitude of soil erosion processes at landscape scale and map it [9]. The RUSLE model is based on the equation:

A=R*K*LS*C*P

where A is the computed average soil erosion rate estimation per unit area (Mg·ha^−1^·yr^−1^), R is the rainfall and runoff erosivity factor (MJ·mm·ha^−1^·h^−1^·yr^−1^); K represent the soil erodibility factor (Mg h·MJ^−1^·mm^−1^); L represents the slope length factor while S, the slope steepness factor; C, the cover-management practice factor, and P, the support practice factor.

To perform RUSLE model we collected and modelled spatial and temporal data for each factor: i) meteorological data based on 20 local weather stations (R factor); ii) pedological data about the erodibility of soils and its susceptibility to erosion (K factor); high resolution topographic data (LS factor); and land use data at regional scale (C factor).

To calculate R values for each of the 20 weather stations (ARPAV) we wrote a specific algorithm run in R software (R Core Team, 2016), performing a intensity rainfall analysis on 10 years of time-series. According to Wishmeier and Smith (1978) and revision by Renard et al. [50] we used the formula that represents the best fit for such regime (S1 Appendix, Fig 1).

**Fig 3:**
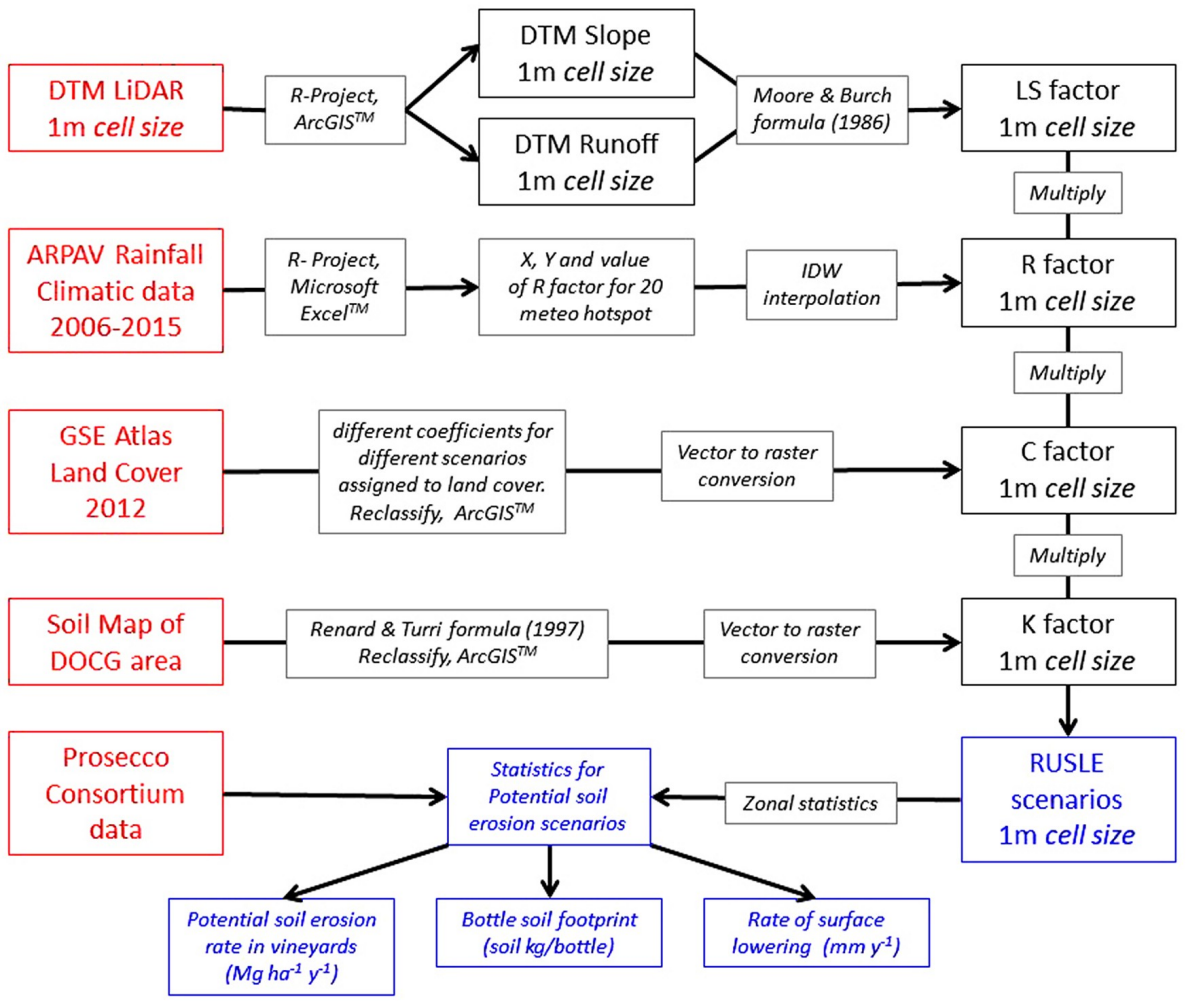
Data input and workflow methodology for soil erosion estimation performed by RUSLE model. In red data inputs; in blue model outputs.

Spatial data for C factor which defines the type of land cover that influences soil erodibility were extracted from the IV Level of CORINE-based dataset at regional scale (2012). To calculate C factor for Prosecco vineyards, we adopted conservative values ranging from 0.12 for conventional vineyards (ARPAV, 2007) to 0.04 for nature-based land management scenario of 100% grassed inter-row vineyard [58]. Input data sources and overall workflow methodology for calculating all RUSLE factors are summarized in Fig 3.

Due to some limitations in estimating LS factor several GIS-modelling techniques were adopted and tested to improve LS estimation [59–62]. To compensate possible overestimation for LS factor we adjusted the slope length (λ), setting the unit contributing area (UCA) on a threshold of 5 ha basin. Therefore, all stream networks greater than 5 ha were automatically excluded for LS calculation, limiting possible overestimations. Moreover, LiDAR DTM at 1-m cell size shows the morphology of all anthropic features, as i.e. terraced landforms and little narrow streets: the slope tool reflects this kind of terrain morphology, reducing all the values along the hillside length by every of each break-slope (S1 Appendix and S2 Fig).

### RUSLE analysis and soil erosion mapping

To perform spatial analysis under conventional land management scenario we analyzed the potential soil erosion in terms of magnitude (Mg ha^-1^ yr^-1^) and total loss (Mg yr^-1^). GIS tools allow not only to estimate potential erosion values but also to display where they are located. Moreover, they provide information about the land use class related to each potential soil erosion value.

We reclassified RUSLE output values in 4 classes: low value (0–4 Mg ha^-1^ yr^-1^), medium value (4–10 Mg ha^-1^ yr^-1^), high value (10–40 Mg ha^-1^ yr^1^), very high value (more of 40 Mg ha^-1^ yr^-1^). By RUSLE analysis we evaluated land use influence on soil erosion phenomenon. We also evaluate the potential soil erosion at Municipality scale in order to highlight the wine-producing district most exposed to potential erosion processes.

After total loss estimation (Mg yr^-1^) in Prosecco vineyards, we estimated what could be the impact in terms of potential erosion on a single bottle of sparkling wine (0.75 L), by aiming to calculate a sort of “soil footprint” for wine production. To do that we analyzed the actual trend of official production of bottled Prosecco DOCG (2015–2018) together with the official policy document for grape production approved by the Veneto Region [43,44].

### Soil erosion under different nature-based land management scenarios

Within the CAP framework, EU promoted the adoption of “best practices” in soil management to control erosion processes by keeping the land under “Good Agricultural and Environmental Condition” (GAEC). Different landscapes features such as grass cover, dry-stone walls, reverse-slope benches on one side, and hedgerows or buffer strips to reduce runoff volume and protect habitats, are included in GAEC standards [12,63]. We therefore performed four different land management scenario simulations at Prosecco DOCG scale, by adopting four different nature-based mitigation measures to increase agricultural sustainability and to protect surface water from loose of herbicides and pesticides: hedgerows, grassed buffer, and a grass cover between inter-rows of vineyards.

In scenario 1 we assigned a conventional grass (C factor 0.005) buffer zone, of 5 m from tail lift of rivers and streams with a minimum value of 2^nd^ order; in scenario 2: we assigned 3.5-m hedgerows of shrub (C factor 0,003) as buffer filter strips around the vineyards; in scenario 3 we modeled a combined scenario summarizing the effects of scenario 1 and scenario 2. Finally, in the fourth one, we simulate a complete greening land management scenario, without the application of herbicides: we simulate to keep grass cover in 100% of vine inter-rows during all seasons. According to Bazzoffi et al. (2017) we used 0.04 value C cover for grassed inter-row vineyard management [58]

## Results

### Soil erosion estimation under conventional land management scenario

**Fig 4:**
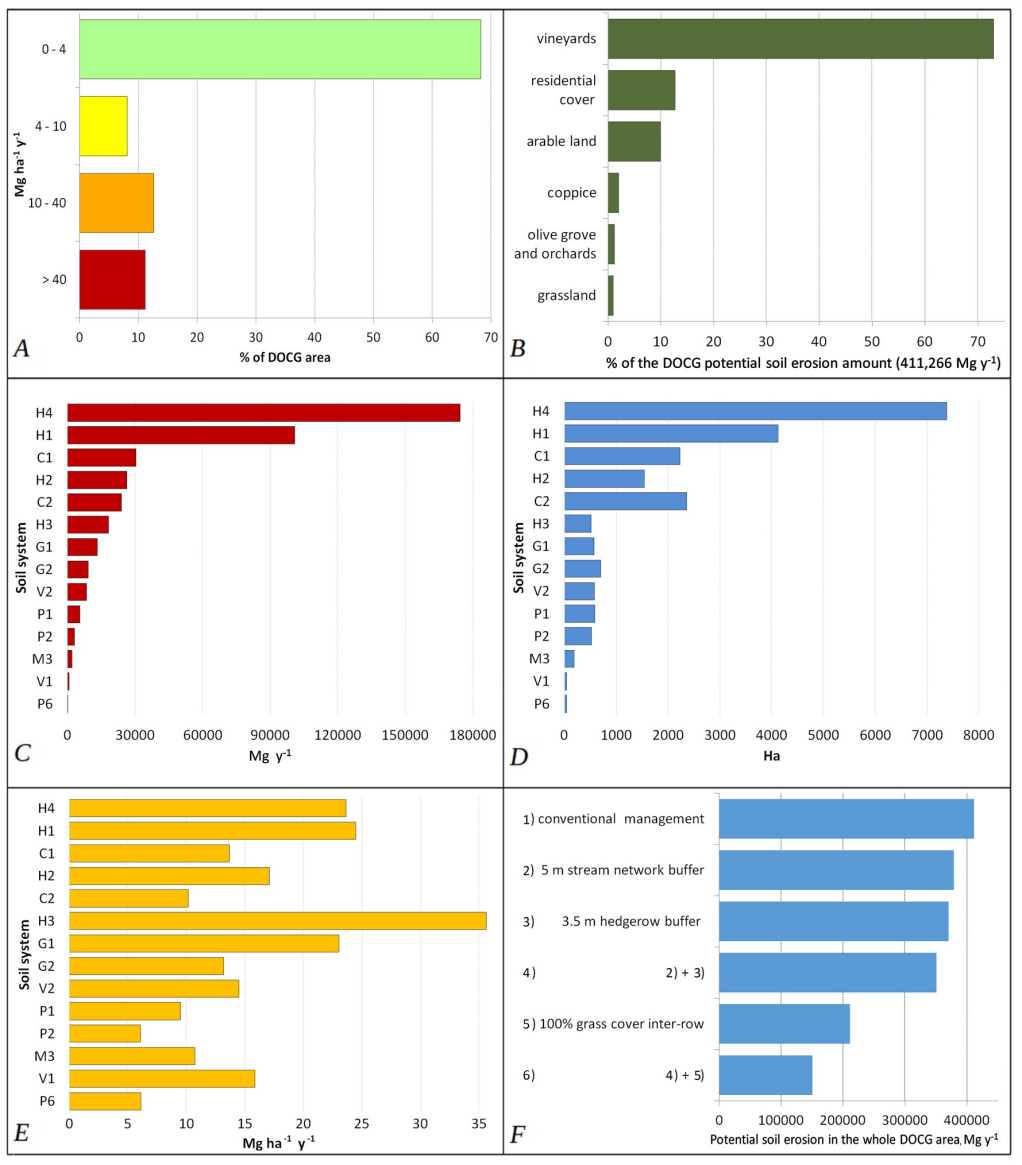
(A) Percentage of the area in RUSLE erosion classes: low erosion (0–4 Mg ha^-1^ yr^-1^), medium erosion (4–10 Mg ha^-1^ yr^-1^), high erosion (10–40 Mg ha^-1^ yr^-1^) and very high erosion (>40 Mg ha^-1^ yr^-1^). (B) Percentage of potential soil erosion from RUSLE modelling in different land use classes. (C) Total Soil erosion estimation in Mg yr^-1^ along the landscapes-soil units (See Table 1). (D) Landscapes-soil units (See Table 1) and surfaces (ha) in the Prosecco DOCG. (E) Soil erosion rate estimation in Mg ha^-1^ yr^-1^ along the landscapes-soil units (See Table 1). (F) Soil erosion estimation in the six different land-management scenarios.

RUSLE analysis showed that the total soil erosion estimation for the Prosecco DOCG area can reach 411.266 Mg yr^-1^, by a rate of 19.5 Mg ha yr^-1^ on average. Beyond this, more of 70% of the total surface showed a potential soil erosion between 0 and 4 Mg ha^-1^ yr^-1^, 12% is between 10 and 40 Mg ha^-1^ yr^-1^, while more than 11% is more 40 Mg ha^-1^ yr^-1^ (Fig 4a).

**Fig 5:**
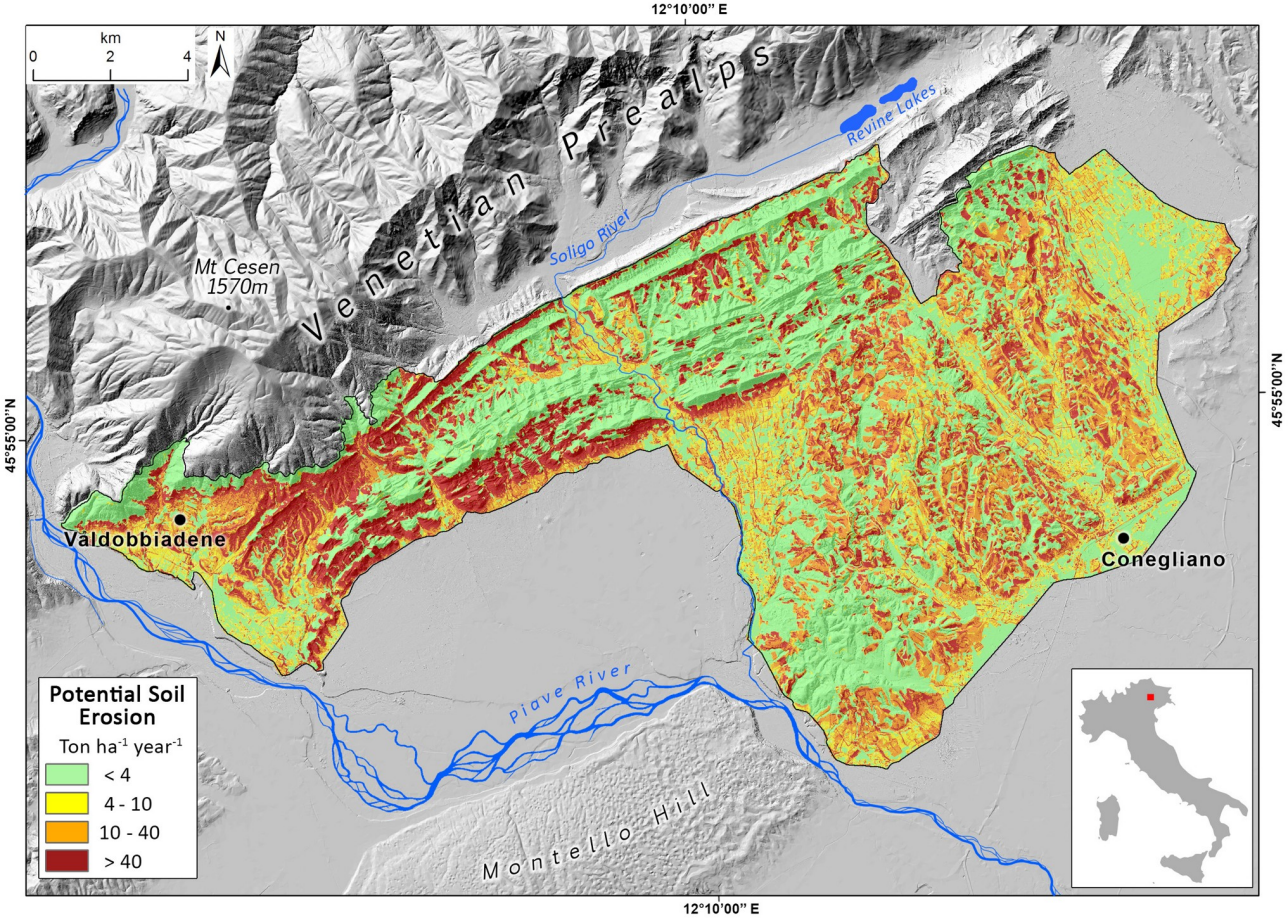
Map of potential soil erosion rate in the Prosecco DOCG area represented in four classes.

The model shows zones with low values near to 0 Mg ha^-1^ yr^-1^ mainly in the gravelly alluvial plain, grassland, forests or slope near 0° degrees; conversely, the highest potential erosion rate values (more of 400 Mg ha^-1^ yr^-1^) are distributed on steepest slopes, mostly on bare soil areas. Generally, potential erosion rate with higher intensity (>40 Mg ha^-1^ yr^-1^) is clustered on long and steep slopes, characterized by agricultural activities (Fig 5). Here, specific land use determines different effects on potential soil erosion rate: olive groves (68.9 Mg ha^-1^ yr^-1^), vineyards (43.7 Mg ha^-1^ yr^-1^), “other permanent crops” (43.9 Mg ha^-1^ yr^-1^). As expected, potential soil erosion rate is more intensive on hilly landscapes, characterized by vineyards cropland, as it shown in Figs 4b, 4c and 5.

If we consider the total soil erosion modelled for all the Prosecco DOCG area, the RUSLE analysis shows that vineyards could reach 300,000 Mg yr^-1^, which contributes to the 73% of all the erosion potential in the whole area (Fig 4b). Assuming an average soil bulk density of 1.3–1.5 g cm^-3^ soil lowering in Prosecco vineyards could reach 3.3–2.9 mm yr^-1^. Therefore, if the average of the declared wine production in the last years is about 90 M bottles, a single bottle of Prosecco DOCG sparkling wine may embody a “soil footprint” on the territory of about 3.3 kg yr^-1^ (Table 2).

**Table 2:** Estimation of potential soil erosion in different land management scenarios and metric units.

Scenarios Class and Units	Scenario 0 conventional vineyards management	Scenario 1 5 m buffer strip around stream network	Scenario 2 3.5 m buffer hedgerow strips around vineyards	Scenario 3 (Scenario 1 + Scenario 2)	Scenario 4 100% grass cover inter-rows	Scenario 5 (Scenario 3 + Scenario 4)
**DOCG surface** **Mg yr^-1^**	411,266	378,410	370,098	350,398	211,330	150,462
**DOCG surface** **Mg ha-1 yr^-1^**	19.5	18.0	17.6	16.6	10.0	7.1
**Vineyards** **Mg yr^-1^**	300,183	300,183	300,183	300,183	101,300	101,300
**Vineyards** **Mg ha^-1^ yr^-1^**	43.7	43.7	43.7	43.7	14.6	14.6
**% vineyards** **Mg yr^-1^**	73	73	73	73	47.4	47.4
**Soil lowering in vineyards (bulk density 1.3–1.5 g/cm^3^) in mm yr^-1^**	3.3–2.9	3.4–2.9	3.4–2.9	3.4–2.9	1.1–1.0	1.1–1.0
**Soil footprint kg bottle^-1^ yr^-1^**	3.3	3.3	3.3	3.3	1.1	1.1

### Soil erosion estimation on landscape soil units

In the study area, soil erosion seems to be potentially higher in soil unit systems H4 and H1 which represent soils in hilly landscapes (Fig 4c). In fact, more than the 66% of soil erosion potential is focused in the hilly sector of the Prosecco DOCG area. Furthermore, H1 plus H4 represent more than 51% of all territory surface (Fig 4d).

As it is illustrated in Fig 4e, if we consider only soil erosion potential expressed by hectares (Mg ha^-1^ yr^-1^) the soil system showing the highest values is H3, losing more than 35 Mg ha^-1^ yr^-1^, while H4 potentially lose 23.6 Mg ha^-1^ yr^-1^. However, H3 such as G1 landscape soil units have limited extension in the study area, so that their contribution to total soil erosion is very low (Fig 4D and 4E).

In the Prosecco wine district, the highest soil erosion potential is localized in the municipality of Farra di Soligo which covers about the 6.9% of total Prosecco DOCG. Here, potential total soil erosion is 70,560 Mg yr^-1^ representing about the 17% of potential soil erosion. The municipality of Valdobbiadene shows 16.5% of total erosion, over the 12.5% of DOCG surface. The Conegliano DOCG area contributes to 9.9% of total erosion, with its 41,324 Mg yr^-1^ but over the 43% of total surface. In fact, the mean potential soil erosion per hectare is 13.6 Mg ha^-1^ yr^-1^, while for Farra di Soligo is about 3 times (47.7 Mg ha^-1^ yr^-1^). The Valdobbiadene Municipality shows 25.7 Mg ha^-1^ yr^-1^, while Vidor show a quite high value (38.2 Mg ha^-1^ yr^-1^) but its surface contributes only to the 2.2% of DOCG surface.

### Nature-based land management scenarios

In the first simulated sustainable scenario, 5 m grassed buffer filter-strips modelled around rivers and streams (197 ha) show a total erosion potential of 378,416 Mg yr^-1^, representing a reduction of the 7.9% of soil erosion. In the second scenario, a reduction of 41.167 Mg yr^-1^ (10%) in potential soil erosion rate was obtained by simulating a mitigation measure of 3.5 m of hedgerows around vineyards, accounting for a total of 645 ha. An important reduction in potential soil erosion is obtained by summarizing the mitigation effects of buffer filter-strips, both around the river networks and vineyards plots: soil loss erosion may be reduced of 14.8%, which corresponds to 60,867 Mg yr^-1^ of soil potentially preserved (Fig 3f, Table 2).

However, the most sustainable scenario in our analyses is represented by simulating 100% grass cover in vine inter-rows. In this case, the total potential erosion in the Prosecco DOCG area would be reduced to 207,100 Mg yr^-1^, saving about the 50% of soil. In vineyards a general decrease of almost 3 times (from 300,000 to 101,325 Mg yr^-1^) is also demonstrated, reducing on average the erosion rate from 43.7 to 14.6 Mg ha^-1^ yr^-1^. In this completely greening scenario total erosion related to vine production in the all Prosecco DOCG area is reduced from 73% to 47%. In the greening scenario of 100% grass cover the soil footprint of a bottle of Prosecco would be 1.1 kg yr^-1^.

All land management scenarios simulated by RUSLE modelling and different metrics to represent potential soil loss are summarized in Table 2.

## Discussion

According to estimation by Cerdan et al. (2010), the mean erosion rate in Italy is 2.3 Mg ha^-1^ yr^-1^ [13]; in our study we estimate that in the Prosecco DOCG area the potential erosion rate modelled is 19.5 Mg ha^-1^ yr^-1^, a magnitude of erosion rate which could be, depending on different land-management scenario, up to 8.5 times higher considering the above mentioned study.

Modelled estimations show soil erosion rate 1.3 times higher than in the Aosta valley vineyard plots (NW Italy), in a similar morphological context and agricultural management practices [28]. According to Aiello et al. (2015) [55], which computed a modified RUSLE model for complex terrain (RUSLE3D) along a highly-erodible hilly landscape in Basilicata (Southern Italy), the mean annual soil erosion in the Bradano basin is 31.80 Mg ha^-1^ yr^-1^, which is 1.6 higher than values estimated in the Prosecco DOCG area.

**Fig 6:**
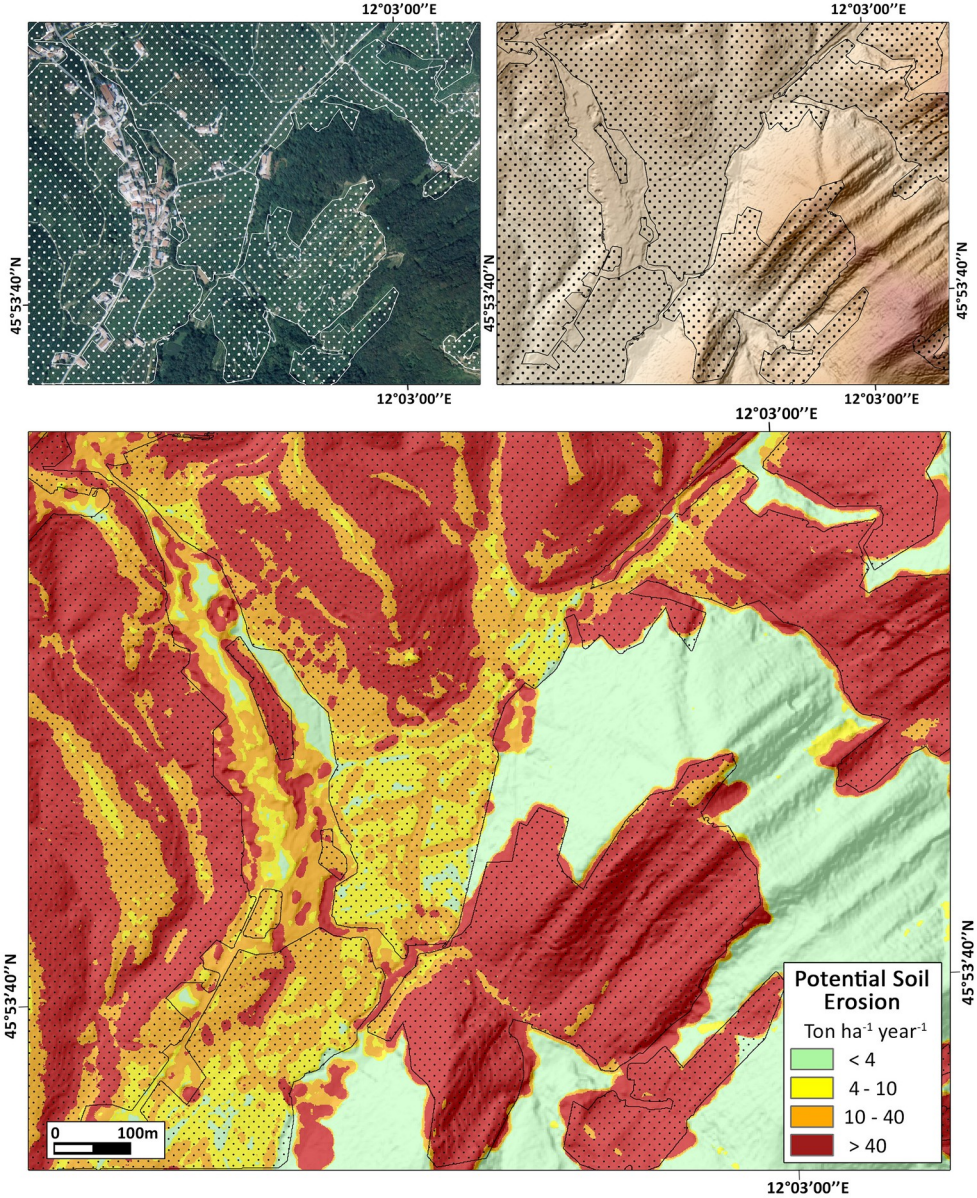
Sample area of S. Stefano di Barbozza (Valdobbiadene Municipality). Upper left: Aerial photo of the village and its surrounding (image modified from “Regione del Veneto—L.R. n. 28/76 Formazione della Carta Tecnica Regionale”). Upper right: DTM of the same area. Lower inset: map of potential soil erosion from RUSLE modelling. Polygons with hatching indicate vineyards.

This study confirmed the key role of vineyards in soil erosion processes, contributing to the highest values (>40 Mg ha^-1^ yr^-1^), mainly clustered in the hilly areas, especially on steep slopes (Figs 5 and 6). This is the case in the areas of Valdobbiadene and Farra di Soligo (Province of Treviso) which account for the 16.5% and the 17% of the total soil erosion in the Prosecco DOCG. The average erosion rate we modelled for conventional land-management scenario in Prosecco vineyards is 43.7 Mg ha^-1^ yr^-1^, which is 31 times higher than the upper limit of tolerable soil erosion threshold defined for Europe by Verhejen et al. (2009) [11]. On the contrary, in the complete greening land management scenario of 100% grass cover the potential erosion rate would be reduced to 10 times the upper limit of tolerable soil erosion threshold. Similar results based on the RUSLE model were found by Prosdocimi et al (2016) in the Lierza river basin of the Prosecco DOCG area [19].

The potential erosion rate estimated in Prosecco DOCG vineyards is quite similar to the ones calculated for the “Chianti Classico” viticultural region (Tuscany, Central Italy), where RUSLE model was validated with field data and measures in 566 experimental sites monitored over six years, and it showed very good performances. The average measured soil loss in “Chianti Classico” vineyards was 42.1 Mg ha^-1^ yr^-1^ against the 43.7 Mg ha^-1^ yr^-1^ modelled for Prosecco DOCG vineyards [26]. However, if we assume a wine production trend close to the limit authorized by the official policy document [44], the soil footprint for the Chianti Classico is about 6.4 kg every single bottle.

Other results performed in experimental plots in sloping vineyards in Germany (Mosel Valley), Eastern Spain (Les Alcusses Valley), and Southwestern Sicily (Agrigento) confirmed soil erosion under conventional land management may range from 19 to 102 Mg ha^-1^ yr^-1^ [21,64–66].

It is worth noting that we performed the most conservative scenario for soil erosion estimation in a conventional vineyard, by using a C standard value of 0.12 for vineyards for RUSLE analyses, not taking into account direct effects of possible new changes to modern agrarian-hydraulic layouts, where terrain morphology and drainage system are strongly modified by vineyards row setup along the steepest slope to facilitate agricultural operations [67,68].

In fact, particular concern is presently given to new vine plantations which are increasing on hillslopes of the Prosecco DOCG area [35,36,46]. As it is widely documented they trigger to extreme erosion rates due to drastic changes in soil physical properties through heavy levelling operations, deep ploughing, trampling, and down-slope orientation of vine-rows [69,70]. Moreover, inter-rows maintenance with bare soil or soil scarcely vegetated by grass cover (5–30%), result in heavy runoff and, therefore, increasing soil erosion rates [27,28]. Different studies highlight that new vine plantations strongly contributes to high erosion risk by increasing rates up to 30 times higher than the upper threshold for tolerable erosion suggested in Europe [11,69,70].

High soil erosion rate may exacerbate in-site effects significantly affecting crop production in soil quality and fertility reduction by decrease in nutrients and organic matter [11,12,53,71]. Moreover, considering the emerging climate change scenarios in Mediterranean regions, an increase in frequency of extreme rainfall events in spring and autumn, especially after dry periods, may amplify off-site impacts on steep slopes by soil water erosion and heavy runoff [72–75]. Off-site impacts are related to non-point source pollution from agricultural fields: pesticide and fertilizers runoff into stream and river network, contamination of groundwater resources, and air pollution by emission of greenhouse gasses such as CO_2_, CH_4_ and N_2_O [9,11,76]. This suggest that in mid- long-term degradation in ecosystem functioning could strongly affect agricultural productivity by drastic reduction in nutrients, organic matter, water capacity and biota.

As it is widely recognized soils are the base of a wide-set of ecosystem good and services which are fundamental for human needs: food production, drinking water quality, water purification, hydrogeological risk control, biodiversity and carbon stock shrinkage. Hence, erosion processes directly lead to degradation and loss of ecosystem services, undermining soil sustainability as recognized both in the seven soil functions defined by the European Commission (2006) and the land-related 2030 UN Sustainable Development Goals [40,41]. Moreover, the European Union brought this issue into the current environmental policy agenda by including soil erosion among the eight soil threats listed within the Soil Thematic Strategy of the European Commission (EC, 2006) and in different policy developments such as the Common Agricultural Policy (CAP), Europe 2020, and the 7th Environmental Action Programme [5].

### Walking pathways by nature-based agricultural practices

The four simulations of sustainable land management scenarios which are included in the GAEC standards show that minor variations in land use could significantly change the total soil loss in the study area. The combination of 5 m grassed buffer filter-strips together with 3,5 m of hedgerows around vineyards potentially preserve 60,867 Mg yr^-1^ of soil. Moreover, as reported in an experimental trial performed in the Prosecco DOCG area, hedgerows represent also an effective mitigation measure to reduce up the 95–98% the spray drift effect of pesticide from vineyards [77].

On the other hand, the forth sustainable scenario which simulate to shift from intensive herbicide application to a 100% inter-row vine grass cover during all seasons time demonstrates the potential effectiveness of this nature-based solution, by reducing soil erosion rate within Prosecco DOCG vineyards of 50%. Results of the simulated mitigation effect are not so different from those derived from experimental measures at field-scale in Sicilian vineyards, where different cover crops sowed in vine inter-rows reduced soil erosion by 68% compared with conventional land management [21]. Similar results were also found in Spain through different erosion measures under simulated rainfall where cover crop of *Secale sp*. and *Brachipodium sp*. showed a significant reduction in soil erosion [78,79]; another immediate effectiveness of field-scale mitigation measures is represented by the use of barley straw mulch in Mediterranean vineyards which reduces the median erosion from 2.81 to 0.63 80,458 Mg yr^-1^ [22]. The most effective mitigation effects at field-scale seems to be the use of straw as mulch together with no-tillage strategy which can reduce soil erosion rate of two orders of magnitude [13].

In conventional land management scenario of vineyard croplands such as the Prosecco DOCG, mitigation measures and best management practices should be adopted in the GAEC framework. They also provide economic incentives to farmers which implement in-site measures like hedgerows and/or grassed buffer filter strips, dry-stone walls terraces, contour farming, and strip cropping to control soil erosion processes in vineyard cropland and to reduce off-site impacts.

## Conclusions

The RUSLE model was applied to estimate the potential total soil erosion in the Prosecco DOCG, by identifying the most critical zones and by simulating five different land-management scenarios at landscape scale (Table 2). This is the first estimation of soil erosion performed at Prosecco DOCG scale. This study highlights the role of Prosecco wine production in potentially high soil erosion processes, which can contribute to the 73% of total erosion in the DOCG area, by in-field rate that is about 31 times higher than the upper limit of tolerable soil erosion threshold defined for Europe. This study confirmed the growing concern of scientific community about soil erosion in conventional land management vineyards.

The estimated erosion rate suggests that i) in mid- long-term period degradation in ecosystem functioning could strongly affect agricultural productivity by drastic reduction in nutrients, organic matter, water capacity and biota; ii) off-site effects such as leaching of agricultural pollutants and potential erosion risk may affect at multiple scale in the territory; iii) nature-based land management practices are very performative mitigation measures to control soil erosion processes.

Using a RUSLE GIS-based approach we modelled a sort of “soil footprint” for producing a single bottle of Prosecco DOCG sparkling wine in conventional land management scenario, which is about 3.3 kg of soil every year. On the other hand, nature-based land management scenarios such as the 100% grass cover in inter-rows showed a reduction of the soil footprint to 1.1 kg/bottle.

RUSLE estimation on potential soil erosion in the Prosecco DOCG viticultural area suggests that an integrated and public monitoring system is needed in the area, by implementing direct/indirect field measures with spatial analyses at agricultural landscape scale.

## Supporting information

S1 Appendix Data set and RUSLE calculation. Description of R, LS and K factors.
(DOCX)

S1 Fig R factor map. Map showing R factor values and weather stations in the Prosecco DOCG.
(TIF)

S2 Fig LS factor map. Map showing LS factor values in the Prosecco DOCG: reduction of LS values is visible along terraced landforms, and 0 values along the main stream networks.
(TIF)

## References

[1] FAO. FAOSTAT [Internet]. 2017 [cited 14 Dec 2018]. http://www.fao.org/faostat/en/#data/EL

[2] FAO. The State of Food and Agriculture [Internet]. Livestock in the Balance. 2016. ISBN: 978-92-5-107671-2 I

[3] Stallman HR. Ecosystem services in agriculture: Determining suitability for provision by collective management. Ecol Econ. Elsevier B.V.; 2011;71: 131–139. 10.1016/j.ecolecon.2011.08.016

[4] Tilman D, Cassman KG, Matson PA, Naylor R, Polasky S. Nature01017. 2002;418. 10.1038/nature01014 12167873

[5] Campbell BM, Beare DJ, Bennett EM, Hall-Spencer JM, Ingram JSI, Jaramillo F, et al Agriculture production as a major driver of the earth system exceeding planetary boundaries. Ecol Soc. 2017;22 10.5751/ES-09595-220408

[6] Montgomery DR. Soil erosion and agricultural sustainability. Proc Natl Acad Sci U S A. 2007;104: 13268–72. 10.1073/pnas.0611508104 17686990 PMC1948917

[7] Gabriels D, Cornelis WM. Human-Induced Land Degradation. Land Use, Land Cover And Soil Sciences. 2009 pp. 131–143.

[8] Parras-Alcántara L, Lozano-García B, Keesstra S, Cerdá A, Brevik EC. Long-term effects of soil management on ecosystem services and soil loss estimation in olive grove top soils. Sci Total Environ. Elsevier B.V.; 2016;571: 498–506. 10.1016/j.scitotenv.2016.07.016 27405516

[9] Jahun BG, Ibrahim R, Dlamini NS, Musa SM. Review of Soil Erosion Assessment using RUSLE Model and GIS. J Biol Agric Healthc. 2015;5: 36–47.

[10] Panagos P, Borrelli P, Meusburger K, van der Zanden EH, Poesen J, Alewell C. Modelling the effect of support practices (P-factor) on the reduction of soil erosion by water at European scale. Environ Sci Policy. Elsevier Ltd; 2015;51: 23–34. 10.1016/j.envsci.2015.03.012

[11] Verheijen FGA, Jones RJA, Rickson RJ, Smith CJ. Tolerable versus actual soil erosion rates in Europe. Earth-Science Rev. Elsevier B.V.; 2009;94: 23–38. 10.1016/j.earscirev.2009.02.003

[12] Panagos P, Borrelli P, Poesen J, Ballabio C, Lugato E, Meusburger K, et al The new assessment of soil loss by water erosion in Europe. Environ Sci Policy. Elsevier Ltd; 2015;54: 438–447. 10.1016/j.envsci.2015.08.012

[13] Cerdan O, Govers G, Le Bissonnais Y, Van Oost K, Poesen J, Saby N, et al Rates and spatial variations of soil erosion in Europe: A study based on erosion plot data. Geomorphology. Elsevier B.V.; 2010;122: 167–177. 10.1016/j.geomorph.2010.06.011

[14] Gómez J, Infante-Amate J, de Molina M, Vanwalleghem T, Taguas E, Lorite I. Olive Cultivation, its Impact on Soil Erosion and its Progression into Yield Impacts in Southern Spain in the Past as a Key to a Future of Increasing Climate Uncertainty. Agriculture. 2014;4: 170–198. 10.3390/agriculture4020170

[15] Ramos MI, Feito FR, Gil AJ, Cubillas JJ. A study of spatial variability of soil loss with high resolution DEMs: A case study of a sloping olive grove in southern Spain. Geoderma. Elsevier B.V.; 2008;148: 1–12. 10.1016/j.geoderma.2008.08.015

[16] Cerdà A, Giménez-Morera A, Bodì MB. Soil and water losses from new citrus orchards growing on sloped soils in the western Mediterranean basin. Earth Surf Process Landforms. 2009;34: 1822–1830. 10.1002/esp.1889

[17] Aurand JM. State of the Vitiviniculture World Market. 38th OIV World Congress of vine and wine. 2015 pp. 1–14.

[18] OIV Organisation Internationale de la Vigne et du Vin. State of the vitiviniculture world market [Internet]. 2018 pp. 1–14. http://www.oiv.int/en/technical-standards-and-documents/statistical-analysis/state-of-vitiviniculture

[19] Prosdocimi M, Cerdà A, Tarolli P. Soil water erosion on Mediterranean vineyards: A review. Catena. Elsevier B.V.; 2016;141: 1–21. 10.1016/j.catena.2016.02.010

[20] Rodrigo-Comino J. Five decades of soil erosion research in “terroir”. The State-of-the-Art. Earth-Science Rev. Elsevier; 2018;179: 436–447. 10.1016/j.earscirev.2018.02.014

[21] Novara A, Gristina L, Saladino SS, Santoro A, Cerdà A. Soil erosion assessment on tillage and alternative soil managements in a Sicilian vineyard. Soil Tillage Res. 2011;117: 140–147. 10.1016/j.still.2011.09.007

[22] Prosdocimi M, Jordán A, Tarolli P, Keesstra S, Novara A, Cerdà A. The immediate effectiveness of barley straw mulch in reducing soil erodibility and surface runoff generation in Mediterranean vineyards. Sci Total Environ. Elsevier B.V.; 2016;547: 323–330. 10.1016/j.scitotenv.2015.12.076 26789370

[23] Ramos MC, Jones G V. Relationships between Cabernet Sauvignon phenology and climate in two Spanish viticultural regions: observations and predicted future changes. 2019;

[24] Norbiato D, Borga M, Dinale R. Flash flood warning in ungauged basins by use of the flash flood guidance and model-based runoff thresholds. Meteorol Appl. 2009;16: 65–75. 10.1002/met.126

[25] García-Ruiz JM, Beguería S, Nadal-Romero E, González-Hidalgo JC, Lana-Renault N, Sanjuán Y. A meta-analysis of soil erosion rates across the world. Geomorphology. Elsevier B.V.; 2015;239: 160–173. 10.1016/j.geomorph.2015.03.008

[26] Napoli M, Cecchi S, Orlandini S, Mugnai G, Zanchi CA. Simulation of field-measured soil loss in Mediterranean hilly areas (Chianti, Italy) with RUSLE. Catena. Elsevier B.V.; 2016;145: 246–256. 10.1016/j.catena.2016.06.018

[27] Tropeano D. Rate of soil erosion processes on vineyards in central Piedmont (NW Italy). Earth Surf Process Landforms. John Wiley & Sons, Ltd; 1984;9: 253–266. 10.1002/esp.3290090305

[28] Biddoccu M, Zecca O, Audisio C, Godone F, Barmaz A, Cavallo E. Assessment of Long-Term Soil Erosion in a Mountain Vineyard, Aosta Valley (NW Italy). L Degrad Dev. 2017; 10.1002/ldr.2657

[29] Lasanta T, Arnáez J, Oserín M, Ortigosa LM, Study AC, Viejo C, et al Marginal Lands and Erosion in Terraced Fields in the Mediterranean Mountains Marginal Lands and Erosion in Terraced Fields in the Mediterranean Mountains. 2001;21: 69–76.

[30] Rodrigo Comino J, Iserloh T, Lassu T, Cerdà A, Keesstra SD, Prosdocimi M, et al Quantitative comparison of initial soil erosion processes and runoff generation in Spanish and German vineyards. Sci Total Environ. Elsevier B.V.; 2016;565: 1165–1174. 10.1016/j.scitotenv.2016.05.163 27265730

[31] Pomarici E. Recent trends in the international wine market and arising research questions. Wine Econ Policy. 2016;5: 1–3. 10.1016/j.wep.2016.06.001

[32] Mariani A, Pomarici E, Boatto V. The international wine trade: Recent trends and critical issues. Wine Econ Policy. 2012;1: 24–40. 10.1016/j.wep.2012.10.001

[33] Varotto M, Tress M. Paesaggi in movimento: il difficile equilibrio tra permanenze e trasformazioni in Valsana. Esercizi di paesaggio. Regione del Veneto, Direzione Urbanistica e Paesaggio; 2011. pp. 111–124.

[34] Visentin F, Vallerani F. A Countryside to Sip : Venice Inland and the Prosecco ‘ s Uneasy Relationship with Wine Tourism and Rural Exploitation. Sustainability. 2018; 2195 10.3390/su10072195

[35] ISPRA Istituto Superiore per la Protezione e la Ricerca Ambientale. Monocolture agricole e degrado del suolo. Considerazioni a partire dal caso dei territori di produzione del Prosecco [Internet]. ISPRA. Consumo di suolo, dinamiche territoriali e servizi ecosistemici. Roma; 2018. http://www.isprambiente.gov.it/it/pubblicazioni/rapporti/consumo-di-suolo-dinamiche-territoriali-e-servizi-ecosistemici.-edizione-2018?set_language=it

[36] Tomasi D, Gaiotti F, Jones G V. The power of the terroir: The case study of prosecco wine. The Power of the Terroir: The Case Study of Prosecco Wine. 2013 10.1007/978-3-0348-0628-2_1

[37] Onofri L, Boatto V, Bianco AD. Who likes it “sparkling”? An empirical analysis of Prosecco consumers ‘ profile. 2015; 10.1186/s40100-014-0026-x

[38] Vinitaly. Il Prosecco Docg cresce più in valore che in volum—Vinitaly [Internet]. [cited 14 Jul 2017]. http://www.vinitaly.com/it/news/wine-news/il-prosecco-docg-cresce-piu-in-valore-che-in-volum/

[39] Distretto del Conegliano Valdobbiadene Centro Studi Rapporto Annuale 2014 Conegliano; 2014.

[40] Distretto del Conegliano Valdobbiadene Centro Studi. Rapporto annuale 2015 [Internet]. Conegliano; 2015. http://www.prosecco.it/wp-content/uploads/2015/06/2015-rapporto-4A.pdf

[41] Distretto del Conegliano Valdobbiadene Centro Studi. Rapporto annuale 2016 [Internet]. Conegliano; 2016. http://www.prosecco.it/wp-content/uploads/2015/06/2016rapporto_annualeConeglianoValdobbiadene.pdf

[42] Distretto del Conegliano Valdobbiadene Centro Studi. Rapporto annuale 2017 [Internet]. Conegliano; 2017. http://www.prosecco.it/wp-content/uploads/2015/06/coneglianovaldobbiadene_rapporto-economico-2017.pdf

[43] Emanuele Scarci. La crescita record del Prosecco. Il Sole 24 ORE. 21 Dec 2016. http://www.ilsole24ore.com/art/impresa-e-territori/2016-12-20/la-crescita-record-prosecco-143556.shtml?uuid=AD994JHC&refresh_ce=1. Accessed 13 Jul 2017.

[44] Regione Toscana. Disciplinare di produzione della Denominazione di Origine Controllata e Garantita dei Vini “Chianti Classico” [Internet]. 2010 [cited 19 Feb 2019] pp. 1–9. http://catalogoviti.politicheagricole.it/scheda_denom.php?t=dsc&q=1023

[45] Bortot F, Iseppi L, Chang TFM, Taverna M. The Boom of Prosecco District: Image of the Utility of the Useless Between Austerity and Recovery I Proc XVIII—IPSAPA Interdiscip Sci Conf. 2014.

[46] Basso M. Land-use changes triggered by the expansion of wine-growing areas: A study on the Municipalities in the Prosecco’s production zone (Italy). Land use policy. Elsevier; 2019;83: 390–402. 10.1016/j.landusepol.2019.02.004

[47] Venzo S. I depositi quaternari e del neogene superiore nella bassa valle del Piave da Quero al Montello e del paleopiave nella valle del Soligo (Treviso) [Internet]. Padova: Istituti di geologia e mineralogia dell’Università de Padova; 1977. http://www.worldcat.org/title/depositi-quaternari-e-del-neogene-superiore-nella-bassa-valle-del-piave-da-quero-al-montello-e-del-paleopiave-nella-valle-del-soligo-treviso/oclc/66003587

[48] Agenzia Regionale per la Prevenzione e protezione ambientale del Veneto. ARPAV La Carta dei Suoli della Provincia di Treviso. Treviso: Provincia di Treviso; 2008.

[49] Kinnell PIA. Event soil loss, runoff and the Universal Soil Loss Equation family of models: A review. J Hydrol. Elsevier B.V.; 2010;385: 384–397. 10.1016/j.jhydrol.2010.01.024

[50] Renard KG, Foster GR, Weesies Gienn A, Porter Jeffrey p. RUSLE: Revised universal soil loss equation. J Soil Water Conserv. Soil Conservation Society of America]; 1991;46: 30–33. http://www.jswconline.org/content/46/1/30.extract

[51] Wischmeier W, Smith DD, Wischmer WH, Smith DD. Predicting rainfall erosion losses: a guide to conservation planning. US Dep Agric Handb No 537. 1978; 1–69.

[52] Ashiagbor G, Forkuo EK, Laari P, Aabeyir R. Modeling Soil Erosion Using Rusle and Gis Tools. Int J Remote Sens Geosci. 2013;2 Available: www.ijrsg.com 26563073

[53] Prosdocimi M, Cerdà A, Tarolli P. Soil water erosion on Mediterranean vineyards: A review. Catena. Elsevier B.V.; 2016;141: 1–21. 10.1016/j.catena.2016.02.010

[54] Biddoccu M, Ferraris S, Cavallo E, Opsi F, Previati M, Canone D. Hillslope Vineyard Rainfall-Runoff Measurements in Relation to Soil Infiltration and Water Content. Procedia Environ Sci. 2013;19: 351–360. 10.1016/j.proenv.2013.06.040

[55] Aiello A, Adamo M, Canora F. Remote sensing and GIS to assess soil erosion with RUSLE3D and USPED at river basin scale in southern Italy. Catena. Elsevier B.V.; 2015;131: 174–185. 10.1016/j.catena.2015.04.003

[56] Rodrigo Comino J, Iserloh T, Morvan X, Malam Issa O, Naisse C, Keesstra S, et al Soil Erosion Processes in European Vineyards: A Qualitative Comparison of Rainfall Simulation Measurements in Germany, Spain and France. Hydrology. 2016;3: 6 10.3390/hydrology3010006

[57] Panagos P, Borrelli P, Meusburger K, Alewell C, Lugato E, Montanarella L. Land Use Policy Estimating the soil erosion cover-management factor at the European scale. Land use policy. Elsevier Ltd; 2015;48: 38–50. 10.1016/j.landusepol.2015.05.021

[58] Bazzoffi P. Erosione del suolo e sviluppo rurale: fondamenti e manualistica per la valutazione agroambientale [Internet]. Edagricole; 2007 https://www.libreriauniversitaria.it/erosione-suolo-sviluppo-rurale-sostenibile/libro/9788850652280

[59] Panagos P, Borrelli P, Meusburger K. A New European Slope Length and Steepness Factor (LS-Factor) for Modeling Soil Erosion by Water. 2015; 117–126. 10.3390/geosciences5020117

[60] Yang X. Digital mapping of RUSLE slope length and steepness factor across New South Digital mapping of RUSLE slope length and steepness factor across New South Wales, Australia. 2015; 10.1071/SR14208

[61] Zhang H, Wei J, Yang Q, Baartman JEM, Gai L, Yang X, et al Geoderma An improved method for calculating slope length (λ) and the LS parameters of the Revised Universal Soil Loss Equation for large watersheds. Geoderma. Elsevier; 2017;308: 36–45.

[62] Hrabalikova M, Janecek, JANEČEK M. Comparison of Different Approaches to LS Factor Calculations Based on a Measured Soil Loss under Simulated Rainfall. Soil Water Res. 2017;2017: 69–77. 10.17221/222/2015-SWR

[63] Bazzoffi P. Soil erosion tolerance and water runoff control: minimum environmental standards. Reg Environ Chang. Springer-Verlag; 2009;9: 169–179. 10.1007/s10113-008-0046-8

[64] Rodrigo Comino J, Quiquerez A, Follain S, Raclot D, Le Bissonnais Y, Casalí J, et al Soil erosion in sloping vineyards assessed by using botanical indicators and sediment collectors in the Ruwer-Mosel valley. Agric Ecosyst Environ. Elsevier B.V.; 2016;233: 158–170. 10.1016/j.agee.2016.09.009

[65] Cerdà A, Keesstra SD, Rodrigo-Comino J, Novara A, Pereira P, Brevik E, et al Runoff initiation, soil detachment and connectivity are enhanced as a consequence of vineyards plantations. J Environ Manage. 2017;202: 268–275. 10.1016/j.jenvman.2017.07.036 28735211

[66] Rodrigo-Comino J, Cerdà A. Improving stock unearthing method to measure soil erosion rates in vineyards. Ecol Indic. Elsevier; 2018;85: 509–517. 10.1016/j.ecolind.2017.10.042

[67] Ramos MC, Porta J. Analysis of design criteria for vineyard terraces in the mediterranean area of North East Spain. Soil Technol. 1997;10: 155–166. 10.1016/S0933-3630(96)00006-2

[68] Martínez-Casasnovas JA, Ramos MC, Ribes-Dasi M. On-site effects of concentrated flow erosion in vineyard fields: Some economic implications. Catena. 2005;60: 129–146. 10.1016/j.catena.2004.11.006

[69] Rodrigo-Comino J, Novara A, Gyasi-Agyei Y, Terol E, Cerdà A. Effects of parent material on soil erosion within Mediterranean new vineyard plantations. Eng Geol. Elsevier; 2018;246: 255–261. 10.1016/j.enggeo.2018.10.006

[70] Rodrigo-Comino J, Brevik EC, Cerdà A. The age of vines as a controlling factor of soil erosion processes in Mediterranean vineyards. Sci Total Environ. Elsevier B.V.; 2018;616–617: 1163–1173 10.1016/j.scitotenv.2017.10.204 29079086

[71] Maetens W, Vanmaercke M, Poesen J, Jankauskas B, Jankauskiene G, Ionita I. Effects of land use on annual runoff and soil loss in Europe and the Mediterranean Prog Phys Geogr. SAGE PublicationsSage UK: London, England; 2012;36: 599–653. 10.1177/0309133312451303

[72] Capolongo D, Pennetta L, Piccarreta M, Fallacara G, Boenzi F. Spatial and temporal variations in soil erosion and deposition due to land-levelling in a semi-arid area of Basilicata (Southern Italy). Earth Surf Process Landforms. John Wiley & Sons, Ltd.; 2008;33: 364–379. 10.1002/esp.1560

[73] Borga M, Anagnostou EN, Blöschl G, Creutin J-D. Flash flood forecasting, warning and risk management: the HYDRATE project. Environ Sci Policy. 2011;14: 834–844. 10.1016/j.envsci.2011.05.017

[74] Sofia G, Roder G, Dalla Fontana G, Tarolli P. Flood dynamics in urbanised landscapes: 100 years of climate and humans’ interaction. Sci Rep. 2017;7: 40527 10.1038/srep40527 28079147 PMC5228191

[75] Zollo AL, Rillo V, Bucchignani E, Montesarchio M, Mercogliano P. Extreme temperature and precipitation events over Italy: assessment of high-resolution simulations with COSMO-CLM and future scenarios. Int J Climatol. John Wiley & Sons, Ltd; 2016;36: 987–1004. 10.1002/joc.4401

[76] Lollino G, Manconi A, Clague J, Shan W, Chiarle M. Effects of Soil Management on Long-Term Runoff and Soil Erosion Rates in Sloping Vineyards. Engineering Geology for Society and Territory. 2015 pp. 1–572. 10.1007/978-3-319-09300-0

[77] Otto S, Loddo D, Baldoin C, Zanin G. Spray drift reduction techniques for vineyards in fragmented landscapes. J Environ Manage. Elsevier Ltd; 2015;162: 290 298. 10.1016/j.jenvman.2015.07.060 26265598

[78] Ruiz-Colmenero M, Bienes R, Eldridge DJ, Marques MJ. Vegetation cover reduces erosion and enhances soil organic carbon in a vineyard in the central Spain. Catena. Elsevier B.V.; 2013;104: 153–160. 10.1016/j.catena.2012.11.007

[79] Ruiz-Colmenero M, Bienes R, Marques MJ. Soil and water conservation dilemmas associated with the use of green cover in steep vineyards. Soil Tillage Res. Elsevier B.V.; 2011;117: 211–223. 10.1016/j.still.2011.10.004

