# Supplementary material for: Estimation of potential soil erosion in the Prosecco DOCG area (NE Italy), toward a soil footprint of bottled sparkling wine production in different land-management scenarios
Source: PLoS One. 2019 May 1;14(5):e0210922. doi: 10.1371/journal.pone.0210922 (PMC6493712; doi:10.1371/journal.pone.0210922)
Supplement: S1 Appendix — Description of R, LS and K factors. (DOCX) [file pone.0210922.s001.docx]

**Supporting Information**

**Appendix**

**R factor**

R factor represents the energy and ability of the rainfall to erode soil. It is strictly related to the main impulsive rainfall events for a specific region [1]. According to local climatic trends, the availability and the spatial distribution of meteorological data, different empirical formulas to calculate R factor were developed by several researchers. Climatic data from the Regional Agency for Environmental Protection and Prevention of the Veneto (ARPAV, 2012), shows for the Veneto Region typical rainfall of modest intensity distributed during the year in two main peaks: one during autumn and another one in spring. Hence, according to local climatic conditions we used the formula that represents the best fit for such regime, according to Wishmeier and Smith (1987) and revision by Renard *et al.* [2]:

$$R=\frac{1}{n}*\sum_{j=1}^{n} \left[ \sum_{k=1}^{m} \left( E \right)*\left( I_{30})_{k} \right) \right]$$

Where:

*n* represents the number of years considered, that in this study are from 2006 to 2015;

*k* is the rainfall number of half hour events;

*E* is the rainfall energy event estimation;

*I_30_* is the amount of rain in 30 minutes;

*m* is the summarize of every rainfall events for all considered years*.*

*E* is calculated using the following expression:

E = 0.29 * [1-0.72^(-0.05*Im^]

where *Im* is the intensity for 5 minutes of rainfall recorded by 20 local weather stations of ARPAV, distributed on all Region.

To calculate R values for each of the 20 weather stations we wrote a specific algorithm and performed it by using R software (R Core Team, 2016) performing a rainfall analysis on 10 years of time-series. The mean R values for each weather stations were, therefore, spatially interpolated using the Inverse Distance Weighted (IDW) algorithm in GIS environment (.

**S1 Fig. R factor map.** Map showing R factor values and weather stations in the Prosecco DOCG.

**C factor**

C factor defines the type of soil cover that influences soil erodibility. Vegetation or artificial cover reduce the erosion effect. The anthropic pavements are impermeable and immobilize the soil. Vegetation has a double effect: leaves partly intercept rain drops, lowering the rainfall kinetic energy at impact with ground (“spalsh erosion”), roots promote water infiltration in the topsoil, lowering surface runoff.

To calculate the C factor, we used the IV Level of CORINE-based dataset at regional scale [3,4]. There are many studies that use several methods to determinate a suitable C value for different type of land use in different morphoclimatic conditions. We therefore selected C values found in literature that best fit the regional environmental conditions [5,6]. The C factor value for each type of land use is traditionally defined by numerous empirical equations and sample points that consider vegetation characteristics and morphological conditions, as surface roughness and surface cover [2]. Concerning vineyards there are different values for C factor in literature (from 1 for arable land, to 0.02 of olive groves), according to the hydraulic-agrarian layouts, percentage of grass cover between rows, and the different techniques and crop practices adopted [7]. This happens because the viticulture is distributed in different climatic zones, from rainy alpine valleys to semi-desert regions in Mediterranean country. In our study, to calculate RUSLE index we adopted a conservative value of 0.12 for vineyards land use as suggested by ARPAV (2008) [5].

## **LS factor**

LS factor represents the topography (length and slope) that influences soil erosion effectiveness [8]. This factor indicates where erosion may act more aggressively and it includes only topographic variables. Depending on these morphological conditions, especially after intense rainfall events, water can acquire high velocity and energy in order to form streams or erosional channels. In order to calculate LS factor, we performed a DTM analysis over 1 m geometric resolution of Laser Imaging Detection and Ranging data (LiDAR) [9]. The formula used for LS factor estimation is after Moore and Burch (1986):

$$LS= \left( Flow accumulation*\left( \frac{cell size}{22.13} \right) \right)^{0.4}* \left( \frac{\left( sin(slope) \right)}{0.0896} \right)^{1.3}$$

where *Flow accumulation* is calculated for each pixel, as the sum of area that lies upstream of the respective basin. In this work the cell size adopted is 1 m. Slope raster was built by function “terrain” in R raster library (R Core Team, 2016) and represents the slope in degrees.

*Flow accumulation* is obviously very high in correspondence of rivers and streams, which results in high LS values that have an important impact on RUSLE final calculation. As our study focuses on areal soil erosion related to surface runoff and incipient rills and gullies, rather than linear erosion along the river network, we chose to compensate possible overestimation for estimating LS factor we adjusted the slope length (λ setting the unit contributing area (UCA) on a threshold of 5 ha basin. Therefore, all stream networks greater than 5 ha were automatically excluded for LS calculation, limiting possible overestimations. Moreover, LiDAR DTM at 1-m cell size shows the morphology of all anthropic features, as i.e. terraced landforms and little narrow streets: the flow accumulation tool reflects this kind of terrain morphology, reducing all the values along the hillside length by every of each break-slope

**S2 Fig. LS factor map.** Map showing LS factor values in the Prosecco DOCG: reduction of LS values is visible along terraced landforms, and 0 values along the main stream networks.

## **K factor**

K factor represents the erodibility of soils and its susceptibility to erosion. The method used for calculation is the original equation of Wischmeier, described in 1978, as reported in Handbook 703 "Predicting soil erosion by water: a guide to conservation planning with the Revised Universal Soil Loss Equation" [2].

In literature, due to the difficulty in recovering soil data required by the original equation, simplified pedofunctions are available for calculating the K factor. At regional level the K factor was calculated using Renard and Torri simplified function (1997) [2,10]: the former requires only values of sand and silt, the latter requires also organic matter. By comparison of results, it was preferred to use data derived from Wischmeier (1978) [11], that considers various soil characteristics, as shown below:

$$K=\frac{\left[ 2.1*{10}^{-4}*\left( 12-OM \right)*M^{1.14}+3.25*\left( s-2 \right)+2.5*\left( p-3 \right) \right]}{759}$$

where:

OM: percentage of organic matter in topsoil;

M: textural parameter (depending on sand, fine sand and silt percentage);

s: structure class code;

p: profile permeability class code.

The K factor was calculated for each type of soil (soil typological unit) and then spatialized relying on the 1:50,000 soil map, available for the whole DOCG area [12].

**References**

1. Lal, R. *Soil Erosion in the Tropics : Principles and Management*; McGraw-Hill, 1990.

2. Renard, K. G.; Foster, G. R.; Weesies Gienn A; Porter Jeffrey p. RUSLE: Revised Universal Soil Loss Equation. *J. Soil Water Conserv.* **1991**, *46* (1), 30–33.

3. Regione Veneto. *Carta Di Copertura Del Suolo Della Regione Del Veneto. GSE Land–Urban Atlas*; Regione del Veneto, 2012.

4. EEA. The Revised and Supplemented Corine Land Cover Nomenclature. *EEA Tech. Rep. No 40* **2000**, No. 38, 110.

5. Agenzia Regionale per la Prevenzione e protezione ambientale del Veneto. ARPAV. *Valutazione Del Rischio Di Erosione per La Regione Veneto*; 2008.

6. Bazzoffi, P. *Erosione Del Suolo e Sviluppo Rurale : Fondamenti e Manualistica per La Valutazione Agroambientale*; Edagricole, 2007.

7. Bazzoffi, P. Soil Erosion Tolerance and Water Runoff Control: Minimum Environmental Standards. *Reg. Environ. Chang.* **2009**, *9* (3), 169–179. https://doi.org/10.1007/s10113-008-0046-8.

8. D. K. McCool, D. K.; L. C. Brown, L. C.; G. R. Foster, G. R.; C. K. Mutchler, C. K.; L. D. Meyer, L. D. Revised Slope Steepness Factor for the Universal Soil Loss Equation. *Trans. ASAE* **1987**, *30* (5), 1387–1396. https://doi.org/10.13031/2013.30576.

9. Blanos, R.; De cillia, C.; Paganini, P.; Pavan, A.; Sterzai, P.; Pietrapertosa, C.; Vidmar, R.; Creati, N.; Martini, G. *Rilievo Lidar Ed Iperspettrale Della Provincia Di Treviso*; 2009.

10. Torri, D.; Poesen, J.; Borselli, L. Predictability and Uncertainty of the Soil Erodibility Factor Using a Global Dataset. *CATENA* **1997**, *31* (1–2), 1–22. https://doi.org/10.1016/S0341-8162(97)00036-2.

11. Wischmeier, W.; Smith, D. D.; Wischmer, W. H.; Smith, D. D. Predicting Rainfall Erosion Losses: A Guide to Conservation Planning. *U.S. Dep. Agric. Handb. No. 537* **1978**, 1–69. https://doi.org/10.1029/TR039i002p00285.

12. Agenzia Regionale per la Prevenzione e protezione ambientale del Veneto. ARPAV. *La Carta Dei Suoli Della Provincia Di Treviso*; Provincia di Treviso: Treviso, 2008.
