# Supplementary figures and images for: Estimation of potential soil erosion in the Prosecco DOCG area (NE Italy), toward a soil footprint of bottled sparkling wine production in different land-management scenarios
Source: PLoS One. 2019 May 1;14(5):e0210922. doi: 10.1371/journal.pone.0210922 (PMC6493712; doi:10.1371/journal.pone.0210922)

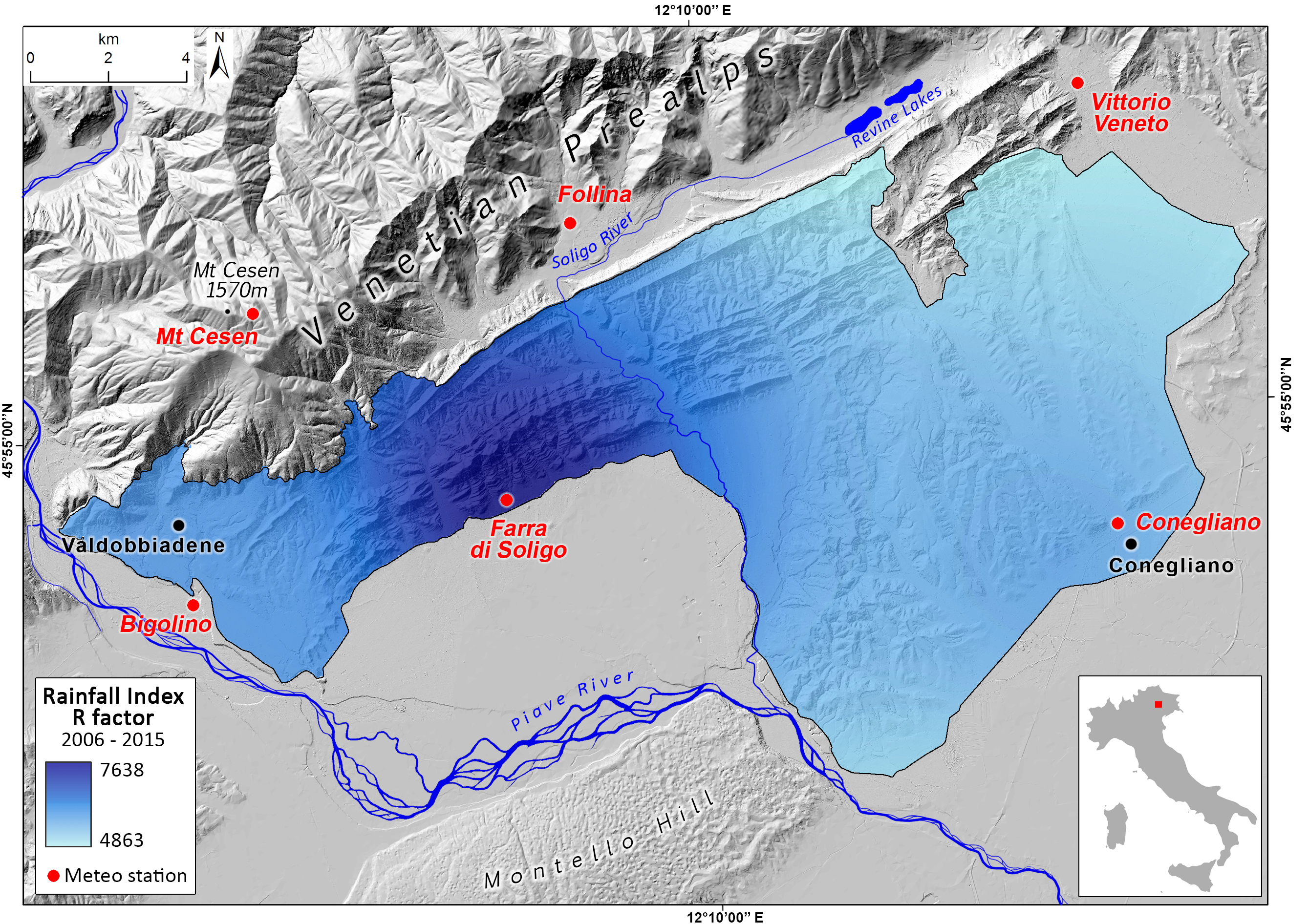

Supplement: S1 Fig — Map showing R factor values and weather stations in the Prosecco DOCG. (TIF) [file pone.0210922.s002.tif]

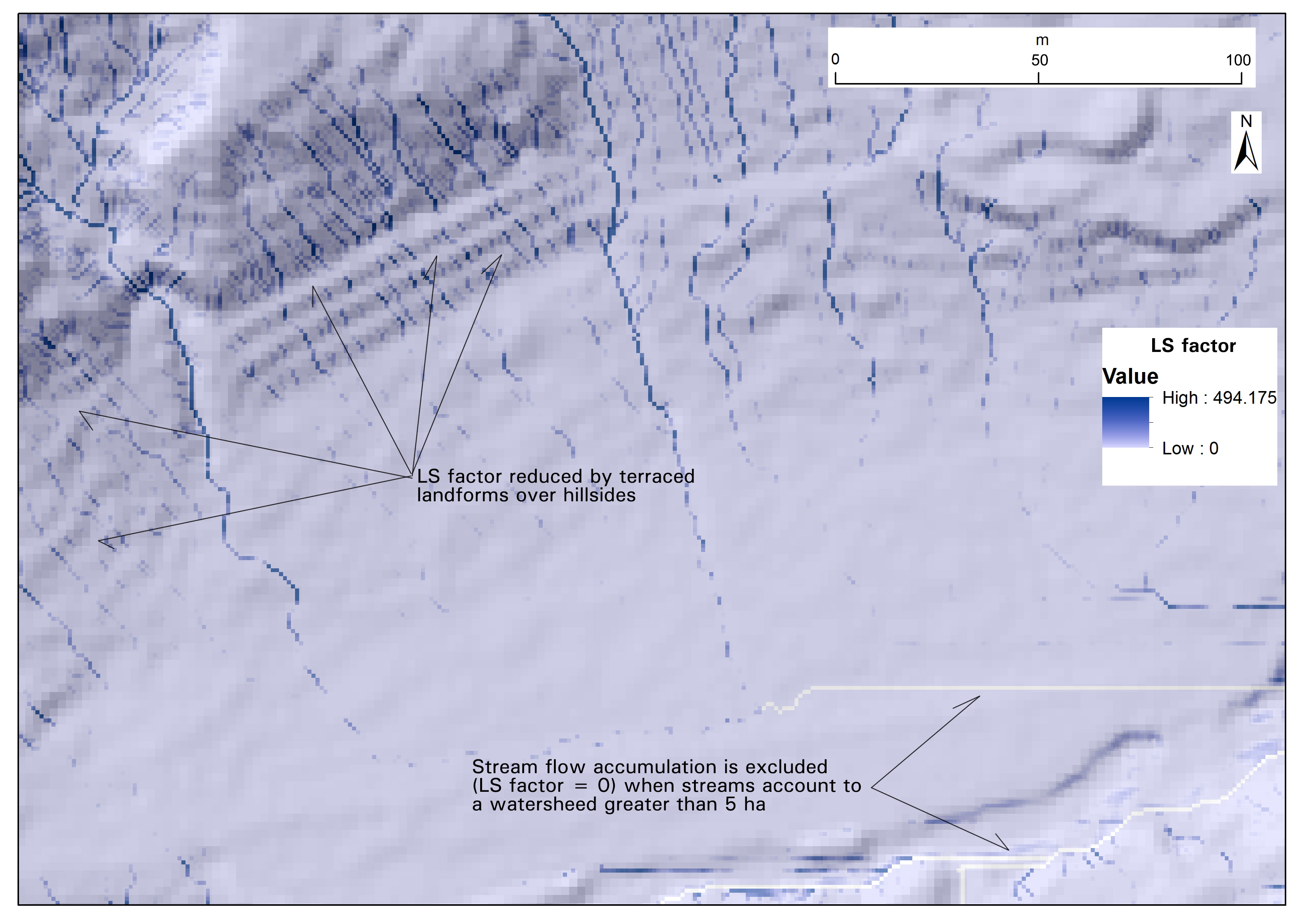

Supplement: S2 Fig — Map showing LS factor values in the Prosecco DOCG: reduction of LS values is visible along terraced landforms, and 0 values along the main stream networks. (TIF) [file pone.0210922.s003.tif]
